# Decreased nocturnal heart rate variability and potentially related brain regions in arteriosclerotic cerebral small vessel disease

**DOI:** 10.1186/s12883-021-02388-1

**Published:** 2021-09-16

**Authors:** Miaoyi Zhang, Huan Yu, Weijun Tang, Ding Ding, Jie Tang, Na Liu, Yang Xue, Xue Ren, Langfeng Shi, Jianhui Fu

**Affiliations:** 1 Department of Neurology, North Huashan Hospital, Fudan University, No.108 Lu Xiang Road, Shanghai, 201900 China; 2 Department of Neurology, Huashan Hospital, Fudan University, No.12 Wulumuqi Zhong Road, Shanghai, 200040 China

**Keywords:** Arteriosclerotic cerebral small vessel disease, Heart rate variability, Nocturnal, Gray matter atrophy

## Abstract

**Background:**

To assess heart rate variability (HRV) among patients with arteriosclerotic cerebral small vessel disease (CSVD) by comparing with control subjects, and to determine whether HRV parameters were related to structural alterations in brain regions involved in autonomic regulation among CSVD patients.

**Methods:**

We consecutively recruited subjects aged between 50 and 80 years who visited the Stroke Prevention Clinic of our hospital and have completed brain magnetic resonance imaging examination from September 1, 2018 to August 31, 2019. Polysomnography and synchronous analyses of HRV were then performed in all participants. Multivariable binary logistic regression was used to identify the relationship between HRV parameters and CSVD. Participants were invited to further undergo three-dimensional brain volume scan, and the voxel based morphometry (VBM) analysis was used to identify gray matter atrophy.

**Results:**

Among 109 participants enrolled in this study, 63 were assigned to the arteriosclerotic CSVD group and 46 to the control group. Lower standard deviation of normal-to-normal intervals (SDNN, OR = 0.943, 95% CI 0.903 to 0.985, *P* = 0.009) and higher ratio of low to high frequency power (LF/HF, OR = 4.372, 95% CI 1.033 to 18.508, *P* = 0.045) during the sleep period were associated with CSVD, independent of traditional cerebrovascular risk factors and sleep disordered breathing. A number of 24 CSVD patients and 21 controls further underwent three-dimensional brain volume scan and VBM analysis. Based on VBM results, SDNN during the awake time (β = 0.544, 95% CI 0.211 to 0.877, *P* = 0.001) and the sleep period (β = 0.532, 95% CI 0.202 to 0.862, *P* = 0.001) were both positively related with gray matter volume within the right inferior frontal gyrus only among CSVD patients.

**Conclusions:**

Decreased nocturnal HRV is associated with arteriosclerotic CSVD independent of traditional cerebrovascular risk factors and sleep disordered breathing. The structural atrophy of some brain regions associated with cardiac autonomic regulation sheds light on the potential relationship.

**Trial registration:**

Trial registration number: ChiCTR1800017902. Date of registration: 20 Aug 2018.

## Introduction

Cerebral small vessel disease (CSVD), a major contributor to stroke and cognitive impairment, is a group of diseases that pathologically affect the small arteries, arterioles, capillaries and venules of the brain [1, 2]. The hallmark neuro-imaging markers of CSVD include recent small subcortical infarcts, lacunes of presumed vascular origin, white matter hyperintensities (WMH) of presumed vascular origin, enlarged perivascular spaces (EPVSs), cerebral microbleeds (CMBs), and brain atrophy [3]. Arteriosclerotic CSVD is one of the most prevalent forms and is strongly associated with aging and hypertension [2]. To date, the pathogenesis of arteriosclerotic CSVD has not been completely elucidated.

Heart rate variability (HRV) is considered to reflect the activity of the autonomic nervous system (ANS) [4]. The relationship between HRV and cardiovascular mortality has been reported in previous epidemiological studies [5, 6]. Recently, accumulating evidence has reported an association between HRV, particularly at nighttime, and the development and presence of subclinical arteriosclerotic CSVD [7, 8]. However, the conclusions on this topic remain controversial. In addition, sleep disorders, particularly sleep disordered breathing (SDB), were not considered as confounding factors in those studies, which in fact are increasingly recognized as risk factors for arteriosclerotic CSVD [9, 10] and affect HRV [11, 12].

The set of brain regions involved in autonomic modulation has been referred to as the central autonomic network (CAN), including the insula, cingulate cortex, medial prefrontal cortex, amygdala, and thalamus [13]. Changes in the morphology of the autonomic circuits have been reported to contribute to a sympathetic-parasympathetic imbalance [14]. Recently, brain atrophy has been considered one of the neuroimaging features of CSVD [3] and is thought to partially mediate the effects of vascular lesions on cognition [15]. Nevertheless, studies on the association between CSVD-related brain atrophy and autonomic dysfunction are scarce.

For these reasons, a plausible hypothesis is that decreased HRV, which is associated with sympathetic overactivity, may be present in patients with arteriosclerotic CSVD, and some structural alterations in cortical regions related to the CAN may play a role in the association. Thus, the current study aimed to assess HRV parameters among patients with arteriosclerotic CSVD and control subjects. The group comparisons were then determined after adjusting for traditional cerebrovascular risk factors and sleep apnea. We further sought to investigate whether the parameters were related to structural alterations in brain regions involved in autonomic regulation.

## Material and methods

### Ethics statement

This study conformed with the World Medical Association Declaration of Helsinki and was approved by the Huashan Hospital Research Ethics Committee (Project-ID: KY2018–224). All patients or their relatives provided written informed consent.

### Subjects

We consecutively recruited subjects aged between 50 and 80 years who visited the Stroke Prevention Clinic of our hospital and have completed brain magnetic resonance imaging (MRI) examination from September 1, 2018 to August 31, 2019. The MRI sequences included T1-weighted, T2-weighted, fluid-attenuated inversion recovery, and susceptibility weighted imaging, using a 3-Tesla scanner (Siemens Magneton Verio 3 T).

The inclusion criteria for patients diagnosed with arteriosclerotic CSVD included 1) baseline MRI scan mainly showing moderate to severe WMH of presumed vascular origin (Fazekas score of 2–3), with or without the presence of other MRI features of CSVD (lacune of presumed vascular origin, CMBs, EPVSs and brain atrophy) [3]; 2) one or more characteristic clinical manifestations of CSVD (including cognitive, motor or mood disturbances) or no evident symptoms; and (3) consent to participate in the study. The exclusion criteria were: 1) cortical infarct or large subcortical infarct (> 2 cm) on conventional MRI; 2) previous ischemic stroke which was less than 6 months after onset; 3) carotid artery stenosis > 50% [16]; 4) non-arteriosclerotic CSVD, such as inherited CSVD or probable cerebral amyloid angiopathy (CAA); 5) any other cause of white matter disease; 6) major psychiatric disorders; 7) the presence of any ANS disorders or clinically relevant arrhythmia; and 8) a systemic or terminal illness that could not complete examinations.

Individuals in the control group were those who visited the Stroke Prevention Clinic with dizziness or headache as chief complaints, but without characteristic neuroimaging markers of CSVD on baseline MRI, except for mild WMH of presumed vascular origin (Fazekas score of 0–1) and age-matched brain atrophy. Other inclusion criteria included: no history of definite cerebrovascular disease, Parkinson’s disease, cognitive impairment or psychiatric disorders; and consent to participate in the study. Enrollment exclusions were: 1) a systemic or terminal illness that could not complete examinations; and 2) the presence of any ANS disorders or clinically relevant arrhythmia.

### Traditional cerebrovascular risk factors

Cerebrovascular risk factors were ascertained through laboratory examinations and interviews conducted by experienced physicians. Hypertension was defined as systolic blood pressure > 140 mmHg or diastolic blood pressure > 90 mmHg, or the use of antihypertensive drugs [17]. Diabetes mellitus (DM) was defined as glycated hemoglobin level > 6.5%, fasting glucose level > 126 mg/dL, 2-h glucose level > 200 mg/dL, or the current use of insulin or hypoglycemic agents [18]. Hyperlipidemia was defined as total serum cholesterol level > 5.9 mmol/L, total triglyceride level > 1.8 mmol/L, or the use of lipid-lowering medications. Previous stroke was defined as the presentation of sudden focal neurological deficits with consistent radiological findings occurred more than 6 months before enrollment. The smoking history (including ex- and current smoker) and body mass index (BMI) were also recorded.

### Polysomnography (PSG)

Eligible subjects in two groups were then referred to the Sleep Center to subsequently complete the PSG examination. Compumedics Profusion PSG V4.5 (Shanghai, China) was used to monitor sleep parameters for a whole night of sleep, and the permission for its application was obtained. Pittsburgh Sleep Quality Index (PSQI) was applied to measure the subjective sleep quality during the last month. All subjects were instructed not to use sleep medications, anxiolytics, or antidepressants for at least 2 weeks preceding the examination and not to consume caffeinated beverages, alcohol or strong tea at the afternoon preceding the recording. The monitoring time ranged from approximately 22:00 pm to 6:30 am the next day and was adjusted according to the individual’s habitual bedtime. Sleep stages, including non-rapid eye movement (NREM) sleep (stage N1, stage N2, stage N3) and rapid eye movement (REM) sleep (stage R), were manually scored by an experienced polysomnographic technologist according to criteria from American Academy of Sleep Medicine [19] (AASM, version 2.4), who was blinded to the medical history of all participants.

Sleep apnea was defined as a ≥ 90% reduction in airflow from baseline lasting more than 10 s (s). Sleep hypopnea was defined as a ≥ 30% reduction in airflow from baseline lasting at least 10 s that was associated with either an oxygen desaturation of > 3% or an arousal. The apnea-hypopnea index (AHI) was defined as the total number of sleep apnea and hypopnea events per hour during the whole night of sleep. AHI in the NREM sleep and REM sleep were also analyzed. Hypoxia-related parameters were also recorded, including the average oxygen saturation (SaO_2_), minimum SaO_2_, average oxygen desaturation, oxygen desaturation index (ODI), time with SaO_2_ < 90% (ST90%), and percentage of cumulative time with SaO_2_ < 90% (CT90%). The ODI referred to the total number of 3% or greater oxygen desaturation events per hour during sleep. The periodic limb movements index (PLMI) was also calculated as the total number of limb movement events per hour during sleep.

### HRV analyses

Electrocardiogram (ECG) data were obtained simultaneously during awake and sleep periods with PSG between the onset and the end of recording. The ECG Add-on for Profusion PSG 4 was applied to analyze the HRV, with a focus on time and frequency domains. Three time-domain measures of HRV were evaluated [11, 20, 21]: the standard deviation of normal-to-normal intervals (SDNN) which characterizes overall HRV, the root mean square of successive differences in RR intervals (RMSSD) and the percentage of normal R-R intervals that differ by 50 ms (PNN50) which are thought to reflect parasympathetic nervous system activity. As a frequency domain measure, the ratio of low to high frequency power (LF/HF) was calculated to estimate the balance between sympathetic and parasympathetic nervous system activity [20, 21]. HRV parameters during awake and sleep periods defined by PSG were recorded and analyzed separately.

### MRI data acquisition and image processing

Based on the MRI results at baseline, all hallmark imaging markers were defined according to the STandards for ReportIng Vascular changes on nEuroimaging (STRIVE) guidelines [3]. The total CSVD scores were calculated according to previous descriptions [22], with a maximum score of four points.

Enrolled patients were invited to further undergo imaging with a three-dimensional brain volume (3D-BRAVO) sequence within 1 week (acquisition parameters: TR = 8.8 ms, TE =1.0 ms, flip angle = 15°, slice thickness = 1 mm iso-voxel, FOV = 320 mm × 320 mm, matrix = 320 × 320, voxel = 1 mm × 1 mm × 1 mm).Voxel-based morphometry (VBM) was implemented using SPM12 on the MATLAB 2016b workstation. The origin of each participant’s image was adjusted to the anterior commissure. Images were segmented into gray matter, white matter, and cerebrospinal fluid using a classical unified segmentation approach with SPM12. Diffeomorphic Anatomical Registration Through Exponentiated Lie algebra (DARTEL) algorithm was used to establish a group-level template for the segmented gray matter images. Then the gray matter images of all subjects were matched to the standard human brain template space of the Montreal Neurological Institute (MNI) using the constructed template. The 3D gray matter images were modulated after high-dimensional registration and then spatially smoothed using a Gaussian filter with a full width at half maximum value of 12 mm to reduce the image noise. Finally, the brain of each subject was divided according to the AnatomyToolbox atlas. The gray matter volume of each brain region was measured quantitatively by calculating the sum of all the voxels classified as gray matter, with voxel size of 1 mm isotropic. Parametric statistical tests were performed for group comparisons of the smoothed gray matter map after including age, sex and AHI as covariates. Differences were considered significant with a family-wise error cluster corrected probability of *p* < 0.0001.

### Statistical analyses

The statistical analyses were performed using SPSS 26.0 and STATA software. The categorical variables were presented as counts and percentages. The continuous variables were presented as medians (interquartile ranges [IQR]). Group comparisons were performed using the chi-squared test for categorical data, Mann–Whitney U test for non-normally distributed continuous data, and t-test for normally distributed continuous data. ﻿The odds ratios (OR) and 95% confidence intervals (CI) for the relationship between HRV parameters and arteriosclerotic CSVD were assessed through multivariate binary logistic regression models. Generalized linear models (GLMs) were applied to analyze the relationships between HRV parameters and gray matter volume in the two groups. Due to the small sample size, variables with a *P* value< 0.05 in the group comparisons were preferentially entered into the models.

## Results

### Patient characteristics

A total of 115 eligible subjects were selected in the current study. Sixty-five patients were diagnosed with arteriosclerotic CSVD, two of whom were excluded because they failed to complete PSG tests. Fifty subjects were assigned to the control group, one of whom was excluded because of severe sleep apnea-hypopnea syndrome (SAHS) with objective cognitive dysfunction and three were excluded because of incomplete PSG records. Thus, 63 patients with arteriosclerotic CSVD and 46 controls were included in the final analysis. The baseline characteristics are summarized in Table 1.

**Table 1:** Baseline characteristics of CSVD and control group

	CSVD group (*n* = 63)	Control group(*n* = 46)	*P*
Sex (male / famale)	41/22	15/31	0.001^*^
Age, years, median (IQR)	68.0 (62.0,73.0)	69.0 (65.0,71.0)	0.455
Hypertension, n(%)	52 (82.5)	8 (17.4)	< 0.001^*^
DM, n(%)	11 (17.5)	4 (8.7)	0.190
History of symptomatic stroke, n(%)	28 (44.4)	0 (0.0)	< 0.001^*^
Hyperlipidemia, n(%)	46 (73.0)	9 (19.6)	< 0.001^*^
Smoking history, n(%)	15 (23.8)	5 (10.9)	0.085
Anti-hypertension drugs, n(%)			
ACEI/ARB	33 (52.4)	5 (10.9)	< 0.001^*^
ß--receptor blocker	11 (17.5)	2 (4.3)	0.037^*^
α-receptor blocker	4 (6.3)	0 (0.0)	0.220
αß-receptor blocker	1 (1.6)	0 (0.0)	1.000
CCB	36 (57.1)	6 (13.0)	< 0.001^*^
diuredics	5 (7.9)	1 (2.2)	0.380
BMI, kg/m^2^, median (IQR)	25.1 (22.2,27.7)	22.3 (20.4,25.4)	0.010^*^
PSQI, median (IQR)	8.0 (4.0,10.0)	7.0 (4.8,10.0)	0.985
Sleep Stage, %, median (IQR)			
Stage N1	8.3 (5.7,14.3)	7.2 (5.2,10.3)	0.064
Stage N2	50.3 (44.4,58.8)	51.3 (45.2,57.5)	0.895
Stage N3	19.8 (11.0,28.4)	20.0 (15.8,26.9)	0.854
Stage R	16.7 (12.1,21.5)	19.0 (16.3,22.2)	0.014^*^
AHI, times/hour, median (IQR)	22.4(12.6, 31.4)	10.8 (6.4,18.2)	< 0.001^*^
AHI in NREM sleep	18.8 (9.4, 30.3)	8.0 (3.6,15.6)	0.004^*^
AHI in REM sleep	36.6 (18.3, 51.9)	23.3 (14.7, 35.0)	< 0.001^*^
Average SaO_2_, %, median (IQR)	94.0 (92.0,95.0)	95.0 (94.0,96.0)	0.005^*^
Minimum SaO_2_, %, median (IQR)	85.0 (81.0,89.0)	89.0 (84.3,90.8)	0.013^*^
Average oxygen desaturation, %, median (IQR)	4.0 (4.0,6.0)	4.0 (4.0,4.8)	0.011^*^
ODI, times/hour, median (IQR)	14.6 (7.4,25.8)	5.7 (2.4,12.4)	< 0.001^*^
ST90%, min, median (IQR)	3.1 (0.3,17.6)	0.8 (0.0,7.3)	0.013^*^
CT90%, %, median (IQR)	1.3 (0.1,7.4)	0.2(0.0,1.8)	0.008^*^
PLMI during sleep period, times/hour, median (IQR)	1.7(0.0,10.1)	1.7(0.0,10.9)	0.933
Average HR during the sleep period, times/min, median (IQR)	67.0 (60.0, 74.0)	62.0 (57.8, 67.3)	0.003^*^
HRV during awake period, median (IQR)			
SDNN	68.7 (54.7,89.4)	73.5 (62.5, 83.3)	0.317
RMSSD	27.2(17.4, 42.4)	30.2(23.9, 43.3)	0.273
PNN50	3.2(0.9,8.9)	5.5(2.8,14.2)	0.059
LF/HF	1.3(0.9,2.1)	1.2(0.8,1.5)	0.081
HRV during sleep period, median (IQR)			
SDNN	53.6 (44.1, 71.9)	70.2 (60.8,77.3)	0.001^*^
RMSSD	26.9 (19.2,40.7)	29.6 (24.1,42.9)	0.304
PNN50	3.3 (1.0,10.2)	6.2 (2.6,14.5)	0.053
LF/HF	1.1 (0.9,2.1)	1.0 (0.7,1.3)	0.006^*^

Statistical significance is reported as * (*P* < 0.05)

Abbreviations: *CSVD* cerebral small vessel disease, *IQR* interquartile range, *DM* diabetes mellitus, *ACEI* angiotensin-converting enzyme inhibitor, *ARB* angiotensin receptor blocker, *CCB* calcium channel blockers, *PSQI* Pittsburgh Sleep Quality Index, *NREM* non-rapid eye movement, *REM* rapid eye movement, *BMI* body mass index, *AHI* apnea- hypopnea index, *SAHS* sleep apnea-hypopnea syndrome, *SaO*_*2*_ oxygen saturation, *ODI* oxygen desaturation index, *ST90%* time with SaO_2_ < 90%, *CT90%* percentage of cumulative time with SaO_2_ < 90%, *PLMI* periodic limb movements index, *HR* heart rate, *SDNN* the standard deviation of normal-to-normal intervals, *RMSSD* the root mean square of successive differences in RR intervals, *PNN50* the percentage of normal R-R intervals that differ by 50 ms, *LF* low-frequency power, *HF* high-frequency power

Sixty-five point one percent (41/63) of patients with arteriosclerotic CSVD (median age 68.0 years) were men, while subjects in the control group (median age 69.0 years) comprised of 32.6%(15/46) men. Difference in the sex composition of the two groups was observed (*P* = 0.001). Significant differences were also noted in the incidence of hypertension, symptomatic stroke and hyperlipidemia in patients with CSVD, as well as the use of anti-hypertension drugs and BMI. Regarding sleep parameters, patients with CSVD showed shorter duration of REM sleep (*P* = 0.014) compared with the control group and showed an overall increase in the AHI, including in the whole night of sleep(*P* < 0.001), NREM sleep(*P* = 0.004) and REM sleep (*P* < 0.001). The group differences in all hypoxia-related parameters also reached statistical significance, including the average SaO_2_, minimum SaO_2_, average oxygen desaturation, ODI, ST90% and CT90%. In addition, age, the incidence of DM, smoking history, PSQI and PLMI were not significantly different between the two groups.

### HRV analyses

A nonparametric test revealed significant differences in the average heart rate (*P* = 0.003), SDNN (*P* = 0.001) and LF/HF (*P* = 0.006) during the sleep period between the two groups, but significant differences were not observed in any of the other parameters (Table 1). The results of binary logistic regression analyses between HRV parameters and arteriosclerotic CSVD are presented in Table 2. After adjusting for sex, hypertension, previous stroke, hyperlipidemia, and BMI (Model 1), patients with arteriosclerotic CSVD had higher LF/HF ratios during both awake (OR = 2.776, 95% CI 1.093 to 7.055, *P* = 0.032) and sleep periods (OR = 5.853, 95% CI 1.626 to 21.063, *P* = 0.007) than the control group. When simultaneously entering multiple variables in Model 1, AHI, ODI and the interaction of the latter two variables (Model 2), significant differences were observed in SDNN (OR = 0.943, 95% CI 0.903 to 0.985, *P* = 0.009) and LF/HF during the sleep period (OR = 4.372, 95% CI 1.033 to 18.508, *P* = 0.045) between the two groups.

**Table 2:** Results of binary logistic regression analyses between HRV parameters and CSVD

	Model 1^a^		Model 2^b^	
	OR (95% CI)	*P*	OR (95% CI)	*P*
Average heart rate during the sleep period, times/min	1.047 (0.975 to 1.124)	0.207	1.023(0.945 to 1.107)	0.577
HRV during awake period				
SDNN	0.994(0.970 to 1.108)	0.624	0.986(0.959 to 1.013)	0.307
RMSSD	1.002(0.980 to 1.025)	0.857	0.995(0.972 to 1.019)	0.693
PNN50	0.981(0.918 to 1.049)	0.581	0.975(0.911 to 1.043)	0.462
LF/HF	2.776(1.093 to 7.055)	0.032^*^	2.053(0.721 to 5.845)	0.178
HRV during sleep period				
SDNN	0.970(0.938 to 1.002)	0.069	0.943(0.903 to 0.985)	0.009^*^
RMSSD	1.003(0.982 to 1.026)	0.758	0.997(0.974 to 1.020)	0.778
PNN50	0.982(0.922 to 1.045)	0.558	0.975 (0.915 to 1.039)	0.430
LF/HF	5.853(1.626 to 21.063)	0.007^*^	4.372(1.033 to 18.508)	0.045^*^

Statistical significance is reported as * (*P* < 0.05)

Abbreviations: *HRV* heart rate variability, *CSVD* cerebral small vessel disease, *BMI* body mass index, *AHI* apnea- hypopnea index, *SDNN* the standard deviation of normal-to-normal intervals, *RMSSD* the root mean square of successive differences in RR intervals, *PNN50* the percentage of normal R-R intervals that differ by 50 ms, *LF* low-frequency power, *HF* high-frequency power

^a^Model 1: adjusted by sex, hypertension, previous stroke, hyperlipidemia, BMI

^b^Model 2: Model 1+ AHI, ODI, AHI × ODI (the interaction item of AHI and ODI)

### Gray matter volume and relationships with autonomic parameters

Complete neuroimaging data were available for only 45 participants: 24 in the arteriosclerotic CSVD group and 21 in the control group. Participant characteristics are presented in Table 3. The median age was 63.5 years in the CSVD group, and 69.0 years in the control group(*P* = 0.014). The incidences of hypertension(*P* < 0.001), previous symptomatic stroke (*P* < 0.001), hyperlipidemia (*P* < 0.001) differed significantly between the two groups. Patients with CSVD showed a higher AHI (*P* = 0.004). Statistical significance existed in SDNN during the sleep period(*P* = 0.009), PNN50 during the awake(*P* = 0.024) and sleep periods(*P* = 0.006) between patients with CSVD and controls. No statistical differences were noted between the two groups in the sex composition, the incidence of DM, smoking history, PSQI, BMI, PLMI during sleep period and other HRV parameters. As for radiologic features, all participants in the control group showed no signs of lacunar infarcts, CMB and moderate to extensive EPVSs in basal ganglia. Among patients with arteriosclerotic CSVD, extensive WMH(91.7%) and the presence of CMB(91.7%) were the most prevalent, followed by moderate to extensive EPVSs in basal ganglia(75.0%) and lacunar infarcts(37.5%). 4(16.7%) patients with arteriosclerotic CSVD had two markers, 10 (41.7%) had three different neuroimaging markers on MRI and all MRI features were simultaneously present in the rest(41.7%).

**Table 3:** Characteristics of subjects included in VBM analyses

	CSVD group (*n* = 24)	Control group(*n* = 21)	*P*
Sex (male / famale)	15/9	8/13	0.102
Age, years, median (IQR)	63.5 (60.8,69.0)	69.0 (68.0,70.0)	0.014^*^
Hypertension, n(%)	21 (87.5)	6 (28.6)	< 0.001^*^
DM, n(%)	5 (20.8)	2 (9.5)	0.527
History of symptomatic stroke, n(%)	11 (45.8)	0 (0.0)	< 0.001^*^
Hyperlipidemia, n(%)	21 (87.5)	4 (19.0)	< 0.001^*^
Smoking history, n(%)	5 (20.8)	1 (4.8)	0.253
Presence of lacunar infarcts, n(%)	9 (37.5)	0 (0.0)	0.006^*^
Extensive WMH^a^, n(%)	22 (91.7)	0 (0.0)	< 0.001^*^
Moderate to extensive EPVSs in basal ganglia^b^, n(%)	18 (75.0)	0 (0.0)	< 0.001^*^
Presence of cerebral microbleeds, n(%)	22 (91.7)	0 (0.0)	< 0.001^*^
Total CSVD burden, n(%)			
1	0 (0.0)	0 (0.0)	–
2	4 (16.7)	0 (0.0)	0.151
3	10 (41.7)	0 (0.0)	0.003^*^
4	10 (41.7)	0 (0.0)	0.003^*^
PSQI, median (IQR)	5.5 (3.0,9.3)	6.0 (4.0,9.0)	0.515
BMI, kg/m^2^, median (IQR)	24.1 (22.3,27.8)	22.4 (20.8,25.4)	0.125
AHI, times/hour, median (IQR)	19.4(12.1, 27.8)	9.5(6.6, 16.6)	0.004^*^
PLMI during sleep period, times/hour, median (IQR)	1.8(0.0, 16.4)	0.4(0.0, 12.4)	0.591
Average HR during the sleep period, times/min, median (IQR)	65.5 (60.8,72.5)	63.0 (57.0,69.0)	0.127
HRV during awake period, median (IQR)			
SDNN	70.7 (61.2, 86.5)	78.5 (64.0, 80.8)	0.585
RMSSD	28.7(19.3, 41.8)	29.7(25.2, 44.3)	0.381
PNN50	3.1(0.9, 9)	7.5(3.8, 16.6)	0.024^*^
LF/HF	1.4(1.0, 2.0)	1.2(0.9, 1.5)	0.231
HRV during sleep period, median (IQR)			
SDNN	53.9 (45.7,64.0)	69.6 (49.4,74.2)	0.009^*^
RMSSD	26.4(19.4, 41.8)	29.3(24.7, 43.5)	0.260
PNN50	2.9(1.0, 9.3)	7.4(4.2, 19.1)	0.006^*^
LF/HF	1.2(0.9, 1.8)	1.0(0.8, 1.2)	0.144

Statistical significance is reported as * (*P* < 0.05)

Abbreviations: *VBM* voxel-based morphometry, *CSVD* cerebral small vessel disease, *IQR* interquartile range, *DM* diabetes mellitus, *WMH* white matter hyperintensity, *EPVSs* enlarged perivascular spaces, *PSQI* Pittsburgh Sleep Quality Index, *BMI* body mass index, *AHI* apnea- hypopnea index, *PLMI* periodic limb movements index, *SDNN* the standard deviation of normal-to-normal intervals, *RMSSD* the root mean square of successive differences in RR intervals, *PNN50* the percentage of normal R-R intervals that differ by 50 ms, *LF* low-frequency power, *HF* high-frequency power

^a^Extensive WMH: deep WMH Fazekas 2–3(confluent or early confluent) and/or periventricular WMH Fazekas 3(extending into the deep white matter)

^b^Moderate to extensive EPVSs in basal ganglia: 10–25 or > 25 EPVSs in basal ganglia

**Fig. 1:**
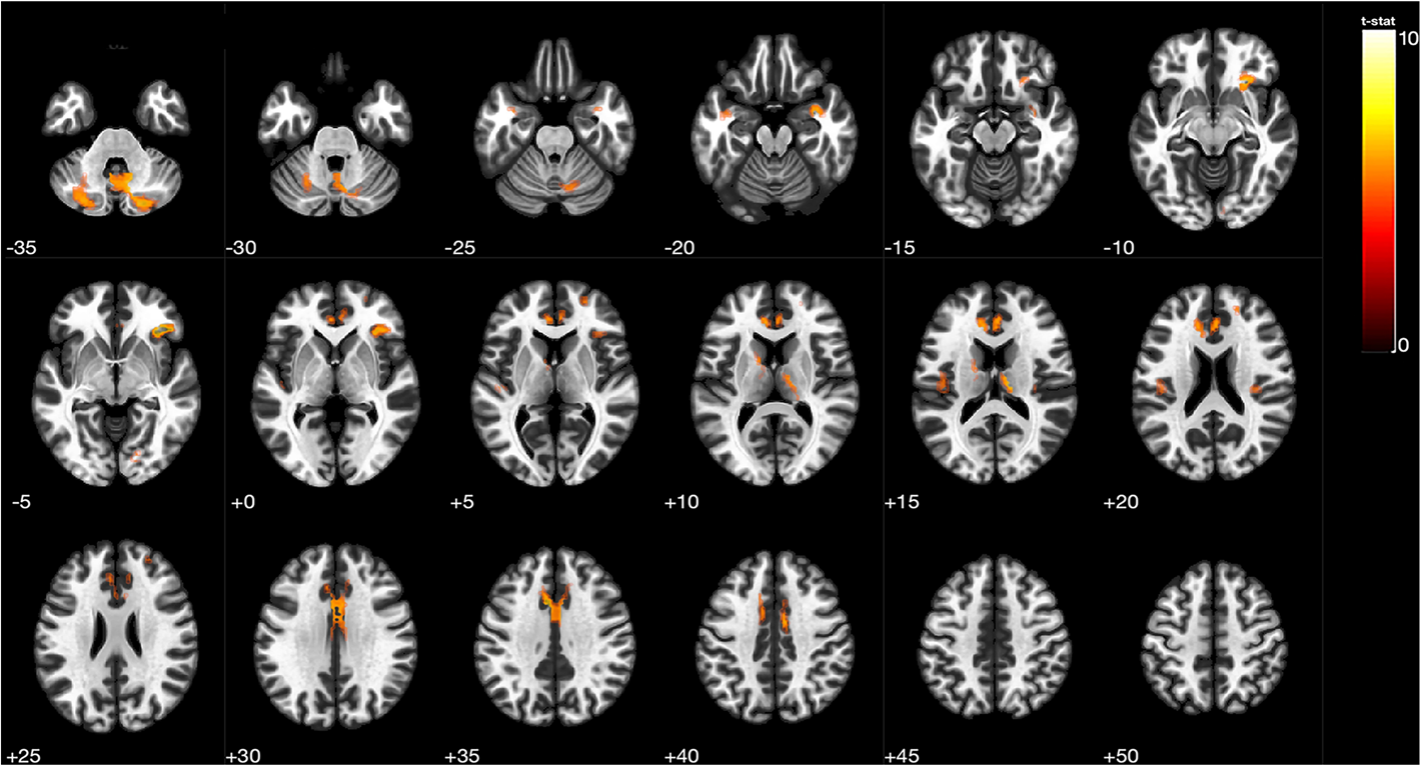
Comparison of gray matter volume between CSVD patients and control participants. The numbers represents the z-axis coordinates of the corresponding plane. Red to yellow represents regions of the brain that have more significant gray matter atrophy in CSVD patients than controls at a threshold of *p* < 0.0001,after adjusting for age, sex and AHI. These brain regions include the bilateral cerebellum, right superior frontal gyrus, right inferior frontal gyrus, right thalamus, right temporal pole, right superior temporal gyrus, right lingual gyrus, right medial cingulate cortex and left anterior cingulate cortex

Using VBM, the arteriosclerotic CSVD group presented with significant gray matter atrophy in certain brain regions, including the bilateral cerebellum, right superior frontal gyrus, right inferior frontal gyrus, right thalamus, right temporal pole, right superior temporal gyrus, right lingual gyrus, right medial cingulate cortex and left anterior cingulate cortex (Table 4 and Fig. 1).GLM analyses were conducted to determine the associations between autonomic parameters and the gray matter atrophy described above in the two groups. Age, AHI, and total burden were entered into the GLMs as covariates. Among patients with arteriosclerotic CSVD, SDNN during the awake and sleep periods were both positively related with gray matter volume within the right inferior frontal gyrus (the former: β = 0.544, 95% CI 0.211 to 0.877, *P* = 0.001; the latter: β = 0.532, 95% CI 0.202 to 0.862, *P* = 0.001). No evident relationship was observed between all parameters and gray matter volume in the control group.

**Table 4:** Regions showing differences in grey matter volume between two groups

Regions^*^	Gray matter volume, mm^3^,median (IQR)		MNI Coordinates			t-value
	CSVD group (*n* = 24)	Control group(*n* = 21)	x	y	z	
Right cerebellum	1.067 (0.958,1.129)	1.153 (1.029,1.240)	17	−57	−50	8.912
Left cerebellum	1.136 (1.050,1.309)	1.270 (1.197,1.458)	−14	−63	−51	8.169
Right inferior frontal gyrus	2.508 (2.228,2.782)	2.497 (2.373,2.658)	33	29	−6	7.405
Right thalamus	4.117 (3.096,4.813)	4.861 (4.407,5.354)	17	−18	15	6.500
Left anterior cingulate cortex.	4.119 (3.637,4.714)	4.643 (4.241,4.908)	−6	6	35	6.492
Right medial cingulate cortex	3.858 (3.440,4.267)	3.858 (3.758,4.142)	9	−9	39	6.155
Right temporal pole	1.394 (1.219,1.467)	1.541 (1.395,1.662)	36	3	−21	6.165
Right superior frontal gyrus	10.320 (9.276,10.893)	9.420 (9.075,9.732)	24	45	23	5.781
Right lingual gyrus	5.929 (5.370,6.444)	6.245 (5.814,6.397)	14	−83	−6	5.594
Left superior temporal gyrus	5.190 (4.761,5.599)	5.046 (4.651,5.551)	−45	−24	5	5.429

*Locations of maximum effect(t > 4.8300; *P* < 0.00001) was shown. Regions were automatically labeled using the AnatomyToolbox atlas. x, y, and z = MNI coordinates in the left-right, anterior-posterior, and inferior-superior dimensions, respectively

Abbreviations: *MNI* Montreal Neurological Institute

## Discussion

The current study demonstrated that patients with arteriosclerotic CSVD show more prominent heart rate fluctuations than controls during the sleep period, independent of traditional cerebrovascular risk factors and SDB, suggesting the existence of sympathetic overactivity. In addition, using quantitative neuroimaging methods, we determined that arteriosclerotic CSVD is accompanied by structural alterations in some brain regions associated with cardiac autonomic regulation.

Regarding HRV measures, SDNN represents overall variability and joint sympathetic and parasympathetic modulation of HRV, while a higher LF/HF ratio indicates a sympathetic predominance. In the current study, lower SDNN and higher LF/HF ratios were observed during sleep among patients with arteriosclerotic CSVD, even after adjusting for SDB, illustrating the potential effect of sympathetic overactivity on the presence of arteriosclerotic CSVD, particularly during the night. Similar to some other reports, the present study did not show a significant difference in HRV during the awake period between the groups [7, 8, 23]. A possible explanation for this finding is that the prominent nocturnal heart rate fluctuations may reflect sustained sympathetic activation and accordingly exert more adverse effects on cerebral white matter [6]. Furthermore, HRV during the awake period is always influenced by many factors, such as physical activity and emotion; thus, measurement bias probably existed [6].

Based on the VBM results, we observed significant reductions in the gray matter volume of certain brain regions among patients with arteriosclerotic CSVD, some of which have been reported to be involved in the central command of cardiac autonomic modulation [14, 24–26]. However, only the right inferior frontal gyrus was shown to be more significantly related to the fluctuations of heart rate during both awake and sleep periods in our study. Interestingly, our results also suggested the preferential atrophy of the right hemispheres in patients with arteriosclerotic CSVD. Right hemispheres have been previously reported to have a dominance of parasympathetic effects [24, 27]. In addition, the current study also suggested significant differences in PNN50, a reflection of parasympathetic function, between patients with arteriosclerotic CSVD and controls included in the VBM analysis. As a result, we posit that pathological changes in the right hemisphere may be associated with the up-regulation of sympathetic tone [27]. Actually, the underlying effects of the aforementioned brain regions on autonomic modulation are complex [14], and the current study failed to detect a loss of gray matter in other vital autonomic regions, such as the insula, hypothalamus and the anterior cingulate cortex. Accordingly, the results from our study should be interpreted with caution, and further studies with a large sample size are still necessary to provide additional evidence.

Due to the design of this study, we have difficulty distinguishing whether the relationships between HRV and arteriosclerotic CSVD are bi-directional or not. Currently, the fluctuations in heart rate induced by elevated sympathetic tone are presumed to increase mechanical shear force on the vessel walls and lead to endothelial injury and arteriosclerosis [28–30]. The ensuing impairment in cerebral autoregulation and the loss of blood-brain barrier integrity likely contribute to the development of arteriosclerotic CSVD [2, 31, 32]. Moreover, brain regions that are already damaged due to vascular deficits are likely to be nonfunctional and therefore have reduced oxygen and perfusion demands, which may accelerate the original ischemic insults and subsequent regional atrophy [33, 34]. Conversely, CSVD-related lesions may occur in fibers of the central autonomic network or contribute to the gray matter loss in brain regions associated with autonomic regulation, which may lead to a secondary imbalance between sympathetic and parasympathetic output. Studies using multimodal neuroimaging examinations are required to further verify the mechanisms described above.

As shown in our study, patients with arteriosclerotic CSVD presented greater fluctuations in nocturnal SaO_2_ than the control group. Due to the significant changes in *P* values after including AHI, ODI and the interaction of both parameters as covariates, a plausible hypothesis is that SDB may be involved in the relationship between HRV and arteriosclerotic CSVD. The precise role is still undetermined. According to recent studies, sympathetic overactivity assessed by monitoring HRV was associated with arteriosclerotic CSVD in patients with obstructive sleep apnea [11, 21]. Furthermore, some scholars have reported an inverse relationship between ODI and the thickness of specific cortical autonomic regions among patients with obstructive sleep apnea, suggesting that intermittent hypoxia and reoxygenation might promote cell apoptosis in some vulnerable regions of the CAN and affect the sympatho-vagal balance [35]. It is noteworthy that although significant differences existed after correcting for SDB, whether CSVD or SDB exerts more effects on HRV still needs to be further investigated.

Our study has certain limitations. The major issue is the unavoidable risk of bias due to the small and unequal sample size of the two groups, which makes multivariate modelling challenging. Moreover, the small sample sizes prevent us from further confirming the associations between HRV and arteriosclerotic CSVD using stratified analyses. Second, patients with arteriosclerotic CSVD and controls were not well gender- matched. Men are previously reported to be at higher risk of stroke and cerebral small vessel disease than women [36, 37], thus the difference in gender in the present study inevitably existed. Given the effect of sex on HRV [38], we have included sex as a covariate in the statistical model, accordingly we consider that the current results could be of some value. Third, heart rate has been reported to differ widely between sleep states, which exhibit a high degree of variability during REM sleep and reach its minimum during NREM sleep [39]. The current study suggested that patients with arteriosclerotic CSVD showed shorter duration of REM sleep and higher AHI throughout the whole night of sleep. Thus we speculate that sleep architecture weights less on the change of nocturnal HRV than the severity of SDB. However, we could not analyze HRV data in different sleep phases and take sleep architecture into account in the multivariable analysis due to the small sample size. Forth, although the current study has suggested a potential association between HRV and CAN among patients with arteriosclerotic CSVD, carotid atherosclerosis is considered another vital cause of the autonomic imbalance [16]. Nevertheless, we excluded patients with carotid artery stenosis > 50% and performed multivariate logistic regression analyses to adjust for traditional cerebrovascular risk factors with a *P* value < 0.05 in the group comparisons. What’s more, a “first night effect” might exist among all participants [40, 41], which might reduce the significance of the difference in HRV between the two groups. Further studies are needed to verify the results.

## Conclusions

In conclusion, the significantly decreased nocturnal HRV is associated with arteriosclerotic CSVD, independent of traditional cerebrovascular risk factors and SDB, suggesting sympathetic overactivity. The structural atrophy of some brain regions associated with cardiac autonomic regulation provides insights into the potential relationship. Prospective studies with larger sample sizes are required to further corroborate the conclusions.

## Data Availability

The datasets used and/or analysed during the current study are available from the corresponding author on reasonable request.

## Abbreviations

CSVD: Cerebral small vessel disease
WMH: White matter hyperintensity
EPVSs: Enlarged perivascular spaces
CMBs: Cerebral microbleeds
HRV: Heart rate variability
ANS: autonomic nervous system
SDB: Sleep disordered breathing
CAN: Central autonomic network
MRI: Magnetic resonance imaging
CAA: Cerebral amyloid angiopathy
DM: Diabetes mellitus
BMI: Body-mass index
PSG: Polysomnography
PSQI: Pittsburgh Sleep Quality Index
NREM: Non-rapid eye movement
REM: Rapid eye movement
AASM: American Academy of Sleep Medicine
AHI: Apnea- hypopnea index
SaO_2_: Oxygen saturation
ODI: Oxygen desaturation index
ST90%: Time with SaO_2_ < 90%
CT90%: Percentage of cumulative time with SaO_2_ < 90%
PLMI: Periodic limb movements index
ECG: Electrocardiogram
SDNN: Standard deviation of normal-to-normal intervals
RMSSD: Root mean square of successive differences in R-R intervals
PNN50: Percentage of normal R-R intervals that differ by 50 ms
LF: Low-frequency power
HF: High-frequency power
3D-BRAVO: Three-dimensional brain volume
VBM: Voxel-based morphometry
SD: Standard deviation
OR: Odds ratio
CI: Confidence interval
GLM: Generalized linear models
SAHS: Sleep apnea-hypopnea syndrome
